# PRKAR2A deficiency protects mice from experimental colitis by increasing IFN-stimulated gene expression and modulating the intestinal microbiota

**DOI:** 10.1038/s41385-021-00426-2

**Published:** 2021-08-04

**Authors:** Lumin Wei, Rongjing Zhang, Jinzhao Zhang, Juanjuan Li, Deping Kong, Qi Wang, Jing Fang, Lifu Wang

**Affiliations:** 1 Department of Gastroenterology, Ruijin Hospital affiliated to Shanghai Jiao Tong University School of Medicine, Shanghai, China; 2 Shanghai Institute for Nutrition and Health, Chinese Academy of Sciences, Shanghai, China; 3 Cancer Institute, The Affiliated Hospital of Qingdao University, Qingdao, China

## Abstract

Protein kinase A (PKA) plays an important role in regulating inflammation via its catalytic subunits. Recently, PKA regulatory subunits have been reported to directly modulate some signaling pathways and alleviate inflammation. However, the role of PKA regulatory subunits in colonic inflammation remains unclear. Therefore, we conducted this study to investigate the role of the PKA regulatory subunit PRKAR2A in colitis. We observed that PRKAR2A deficiency protected mice from dextran sulfate sodium (DSS)-induced experimental colitis. Our experiments revealed that the intestinal epithelial cell-specific deletion of *Prkar2a* contributed to this protection. Mechanistically, the loss of PRKAR2A in *Prkar2a*^−/−^ mice resulted in an increased IFN-stimulated gene (ISG) expression and altered gut microbiota. Inhibition of ISGs partially reversed the protective effects against DSS-induced colitis in *Prkar2a*^−/−^ mice. Antibiotic treatment and cross-fostering experiments demonstrated that the protection against DSS-induced colitis in *Prkar2a*^−/−^ mice was largely dependent on the gut microflora. Altogether, our work demonstrates a previously unidentified function of PRKAR2A in promoting DSS-induced colitis.

## Introduction

Inflammatory bowel disease (IBD) is a group of chronic, relapsing inflammatory disorders of the gastrointestinal tract, which mainly include Crohn’s disease and ulcerative colitis (UC)^1^. Although many factors such as genetic predisposition, epithelial barrier defects, dysregulated immune responses, and gut microbiota dysbiosis have been demonstrated to participate in the occurrence of IBD, its pathogenesis is still not fully understood^2^.

Protein kinase A (PKA) is a silk/threonine protein kinase that acts as a signal transducer by sensing cAMP signaling and catalyzing the phosphorylation of downstream substrate proteins^3^. In the non-activated state, PKA forms a tetrameric protein with a regulatory subunit (PKAR) dimer and two catalytic subunits (PKAC). Four regulatory isoforms, namely, PRKAR1A, PRKAR1B, PRKAR2A, and PRKAR2B, are present, and each is encoded by a separate gene^4,5^. PKA subunits have distinct expression patterns: PRKAR1A and PRKAR2A are ubiquitously expressed, while PRKAR1B and PRKAR2B are expressed principally in the brain and adipose tissues^6–9^. Signaling via PKA has been demonstrated to play an important role in regulating inflammatory conditions, including IBD^10–12^. PKA activation by phosphodiesterase enzyme 4 (PDE4) inhibitors can be useful in alleviating colon inflammation in patients with IBD^13^. However, the Phase II clinical trial of Tetomilast, a PDE4 inhibitor, which was conducted in patients with IBD, reported unsatisfactory results^14^. For many years, the studies on PKA signaling have focused on its catalytic subunits. However, the role of PKA regulatory subunits might have been overlooked. Global knockout of PRKAR2B produced a genetically lean mouse, which is resistant to obesity and exhibits an improved insulin resistance when challenged with a high-fat diet^15,16^. An ex vivo study showed that PRKAR2A and PRKAR2B bind directly to protein Gαi and activate downstream mitogen-activated protein kinase signaling pathways in yeast and HEK293 cells^17^. Additionally, PRKAR2A has been reported to alleviate inflammation in myocardial infarction by directly binding to interferon (IFN)-γR2^18^. Despite the emerging evidence on the importance of PKA regulatory subunits, their role in colon inflammation has not been evaluated till date. Thus, the complete picture of PKA signaling in IBD has not been elucidated. We hypothesized that PRKAR2A, the most abundant PKA regulatory subunit in colon tissues, might participate in regulating colonic inflammation. Therefore, we conducted this study to investigate the role of PRKAR2A in colitis.

In this study, we demonstrated that PRKAR2A phosphorylation (p-PRKAR2A) was decreased in colonic mucosal of patients with UC and in mice with dextran sulfate sodium (DSS)-induced colitis. Global knockout of PRKAR2A alleviated DSS-induced colitis by promoting type I IFN-stimulated gene (ISG) expression and modulating gut microbial composition. The protection against DSS-induced colitis was due to a deficiency of PRKAR2A in intestinal epithelial cell (IEC). Our data elucidates a function of PRKAR2A deficiency in ameliorating DSS-induced colitis, which was earlier unidentified, thus suggesting that PRKAR2A might contribute to the unsatisfactory results of PDE4 inhibitor in the IBD clinical trial.

## Results

### p-PRKAR2A is downregulated in patients with UC and mice with DSS-induced colitis

**Fig. 1:**
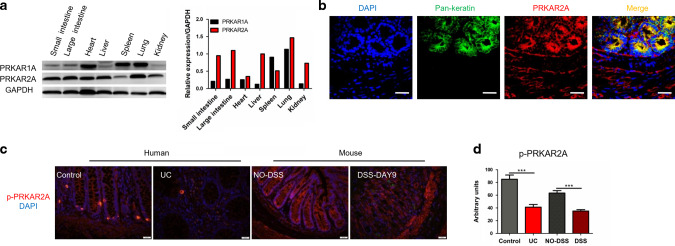
Decreased phosphorylation of PRKAR2A in UC patients and mice with DSS-induced colitis. **a** The expression of PRKAR1A and PRKAR2A in different tissues of WT mice. **b** Colon tissues of WT mice were stained with DAPI (blue), antibody to Pan-keratin (green), and antibody to PRKAR2A (red). Representative immunofluorescent images and merged images are shown. Bar = 20 μm. **c** Representative images of immunofluorescent staining for p-PRKAR2A(ser99) in colon tissues collected from human and mice. Nuclei were counterstained with DAPI. **d** Semi-quantification of the level of p-PRKAR2A in human (UC patients (*n* = 5) and uninflamed controls (*n* = 3)) and mice (treated (*n* = 5) and untreated (*n* = 5) with DSS). Data shown in **a**–**d** are representative of two independent experiments. Data are presented as mean ± SEM. Student’s *t* test was used to do the analysis. ***P < 0.001, bar = 50 μm.

First, we evaluated two widely expressed PKA regulatory subunits (PRKAR1A and PRKAR2A) in different mouse tissues. PRKAR2A was mainly expressed in the intestine, heart, liver, and lungs, whereas PRKAR1A was mainly expressed in the heart, spleen, and lungs (Fig. 1a), suggesting that PRKAR2A is the predominant PKA regulatory subunit in the intestine. To characterize the cellular origin of PRKAR2A in colonic mucosa, colon tissues were immunostained with 4’,6-diamidino-2-phenylindole (DAPI; nuclear marker), anti-Pan-keratin (a marker for IECs), and anti-PRKAR2A antibodies. We found that PRKAR2A was mainly detected in the epithelial regions of colonic mucosa (Fig. 1b). Unlike PRKAR1A, which does not possess a phosphorylation site, PRKAR2A is phosphorylated on Ser99. We observed that PRKAR2A phosphorylation was decreased in the colonic mucosa of patients with UC than in the colonic mucosa of uninflamed donors (Fig. 1c, d). To confirm this result, we administered 2% DSS to wild-type (WT) mice to induce experimental colitis. DSS-induced colitis is a commonly used mouse model that mimics the clinical pathology of IBD. We observed that p-PRKAR2A was downregulated in the colonic mucosa of DSS-treated mice than in the mucosa of untreated littermates (Fig. 1c, d). Taken together, our data reveal that p-PRKAR2A is downregulated in patients with UC and mice with DSS-induced colitis.

### *Prkar2a*^−*/*−^ mice are resistant to DSS-induced colitis

**Fig. 2:**
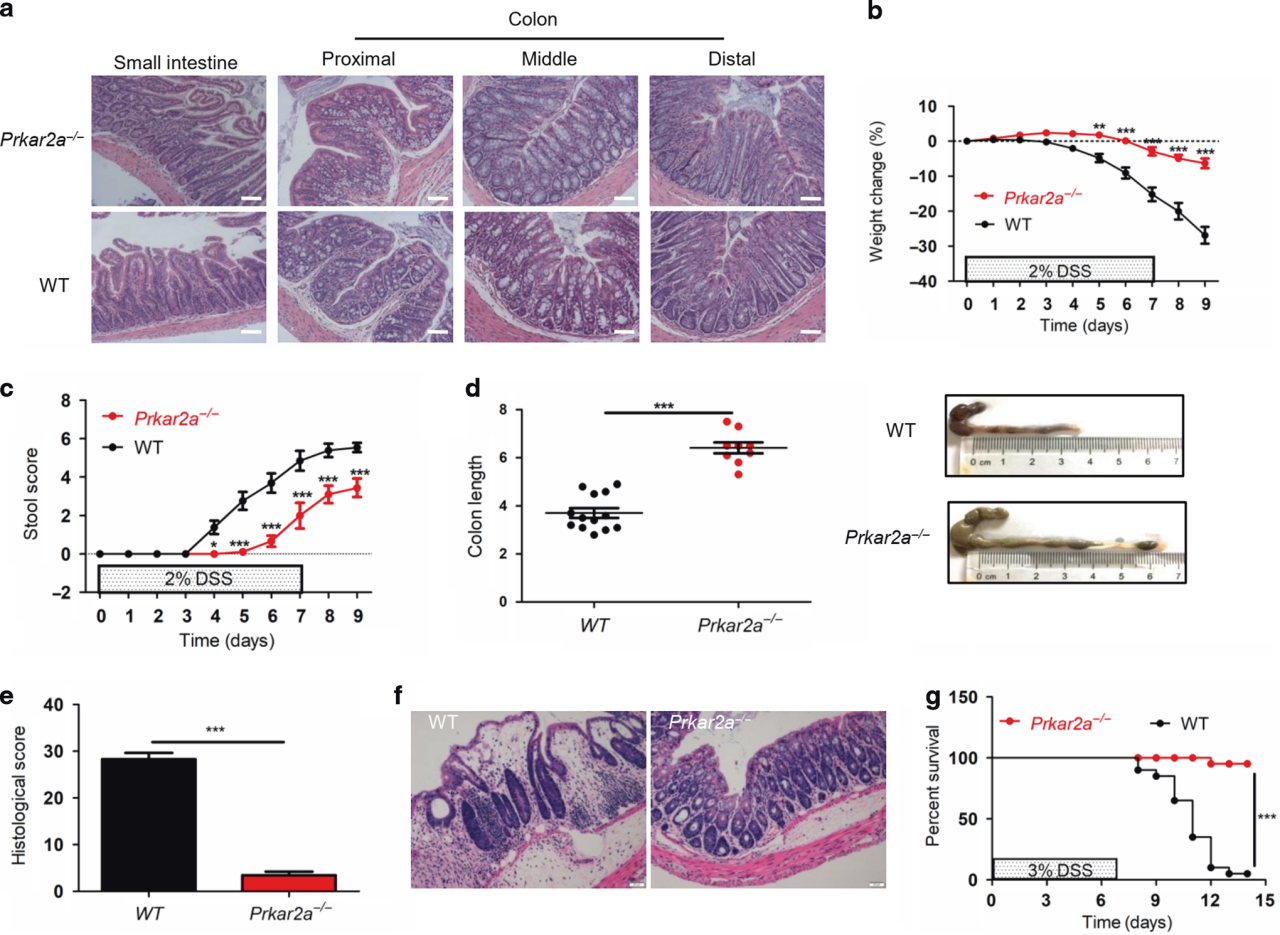
Prkar2a deficiency provides protection against DSS-induced colitis. **a** Representative images of H&E-stained small and large intestine of *Prkar2a*^−/−^ and WT mice. Scale bar = 50 μm. **b**–**f** Colitis was induced in WT (*n* = 13) and *Prkar2a*^−/−^ (*n* = 9) mice by adding 2% DSS in the drinking water for 7 days, followed by 2 days of regular water. **b** Body weight was monitored over 9 days. Graph shows the percentage of body weight relative to initial body weight. **c** Stool score of DSS-treated WT and *Prkar2a*^−/−^ mice were measured every day during colitis development. **d** Colons were removed and colon lengths were determined at day 9. **e** Histological scores of colitis. **f** Representative microscopic images of H&E-stained colons at day 9. Scale bar = 50 μm. **g** Survival curve of the WT (*n* = 20) and *Prkar2a*^−/−^ (*n* = 16) mice challenged with 3% DSS. Log-rank (Mantel–Cox) test was used to do the analysis. ***P < 0.001. Data shown in **a**–**g** are representative of three independent experiments. Data are presented as mean ± SEM. Student’s *t* test (**d**, **e**) or two-way ANOVA (**b**, **c**) was used to compare experimental groups. ***P < 0.001; **P < 0.01; *P < 0.05.

To clarify whether PRKAR2A regulates the development of colitis, we used PRKAR2A-deficient (*Prkar2a*^−*/*−^) mice in this study. Consistent with a previous report^19^, *Prkar2a*^−*/*−^ mice developed normally and showed no spontaneous gut pathology on histological analysis (Fig. 2a). WT and *Prkar2a*^−*/*−^ mice were subjected to DSS treatment, and they were monitored for clinical signs of gastrointestinal disease, such as weight loss, diarrhea, and rectal bleeding. Stool consistency and rectal bleeding were monitored as stool score. After treatment with 2% DSS for 7 days, followed by 2 days of administering regular water alone, *Prkar2a*^−*/*−^ mice exhibited a significantly decreased body weight loss (Fig. 2b), lower stool score (Fig. 2c), and less colon shortening (Fig. 2d) than their WT counterparts. Histological analysis of hematoxylin and eosin (H&E)-stained colon tissues revealed significantly lower histological scores in *Prkar2a*^−*/*−^ mice than in WT mice (Fig. 2e), as demonstrated by a decreased epithelial/crypt damage and leukocyte infiltration in *Prkar2a*^−*/*−^ mice (Fig. 2f). To further determine the protective effect of *Prkar2a* deficiency in DSS-induced colitis, we increased the concentration of DSS to 3% and extended the observation time to 14 days. The mortality of *Prkar2a*^−*/*−^ mice was significantly lower than that of WT mice after treatment with 3% DSS (Fig. 2g). Taken together, our results suggest that the absence of PRKAR2A protects mice from DSS-induced colitis.

### Genetic ablation of PRKAR2A in IECs ameliorates DSS-induced colitis

**Fig. 3:**
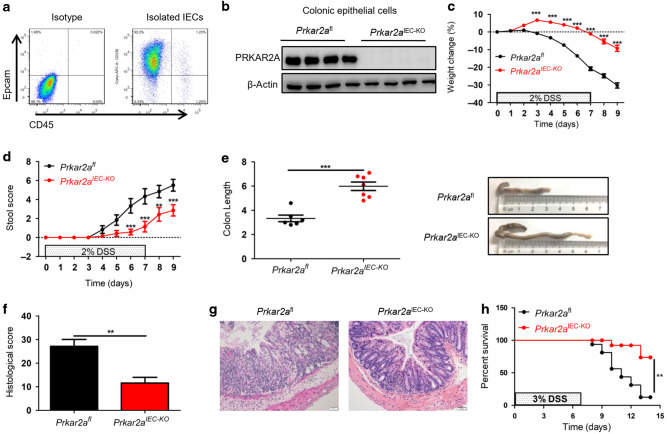
Ablation of *Prkar2a* in IECs protects against DSS-induced colitis. **a** Flow cytometric analysis of Epcam+CD45− intestinal epithelial cell frequencies in colonic epithelial cells isolated from *Prkar2a*^IECKO^ mice. Cells were gated on Epcam and CD45. **b** Colonic epithelial cells from *Prkar2a*^IEC-KO^ mice and *Prkar2a*^fl^ mice were collected and subjected for western blot with PRKAR2A antibody. Experiments in **a**, **b** were repeated at least three times. **c**–**g** *Prkar2a*^fl^ (*n* = 6) and *Prkar2a*^IEC-KO^ (*n* = 7) mice were treated with 2% DSS for 1 week followed by 2 days normal drinking water. **c** Weight loss was monitored daily and is displayed as the percentage of the initial body weight. **d** Stool score was measured every day during colitis development. **e** At day 9, colons were removed and colon lengths were determined. **f** Histological analysis of distal colon tissues at day 9 of experimental colitis. **g** Representative images of distal colon at day 9. Scale bar = 50 μm. **h** The survival curve of *Prkar2a*^fl^ (*n* = 16) and *Prkar2a*^IEC-KO^ (*n* = 12) mice. Mice were treated with 3% DSS for 1 week and the mortality was monitored over 14 days. Log-rank (Mantel–Cox) test was used to do the analysis. Data shown in **c**–**h** are representative of three independent experiments. All graphs show mean ± SEM. Student’s *t* test (**e**, **f**) or two-way ANOVA (**c**, **d**) was used to compare experimental groups. ***P < 0.001; **P < 0.01.

As PRKAR2A was mainly detected in IECs, we investigated whether the protection against DSS-induced colitis in *Prkar2a*^−*/*−^ mice was due to an intrinsic epithelial PRKAR2A deficiency. *Prkar2a*^fl^ mice were crossed with Villin-Cre transgenic mice^20^ to generate mice with an IEC-specific deletion of *Prkar2a* (*Prkar2a*^IEC−KO^), where Cre-mediated deletion of *Prkar2a* was restricted to the large and small intestines. As predicted from global *Prkar2a* knockout mice, *Prkar2a*^IEC−KO^ mice were viable and did not show any intestinal abnormalities on histological examination (data not shown). To confirm the deletion efficiency, we isolated colonic epithelial cells and analyzed their purity using flow cytometry (Fig. 3a). As expected, PRKAR2A was efficiently ablated in the colonic epithelium of *Prkar2a*^IEC−KO^ mice (Fig. 3b). To investigate the functional role of epithelial intrinsic PRKAR2A in colitis development, *Prkar2a*^IEC−KO^ and control (*Prkar2a*^fl^) mice were challenged with 2% DSS for 7 days followed by 2 days of recovery with normal drinking water. Similar to *Prkar2a*^−/−^ mice, *Prkar2a*^IEC−KO^ mice showed significantly slower rate of weight loss (Fig. 3c), lower stool score (Fig. 3d), and less colon shortening (Fig. 3e) than *Prkar2a*^fl^ mice. These findings were confirmed via histopathological analysis of H&E-stained colon tissues (Fig. 3f). *Prkar2a*^IEC−KO^ mice exhibited less severe epithelial/crypt erosion and decreased numbers of infiltrating mucosal and submucosal leukocytes than *Prkar2a*^fl^ mice (Fig. 3g). The survival experiment showed that the mortality of *Prkar2a*^IEC−KO^ mice was significantly lower than that of *Prkar2a*^fl^ controls after 3% DSS administration (Fig. 3h). Collectively, our results indicate that IECs contribute to the protection against DSS-induced colitis in *Prkar2a*^−*/*−^ mice.

### Myeloid cell-specific deletion of *Prkar2a* does not ameliorate DSS-induced colitis

**Fig. 4:**
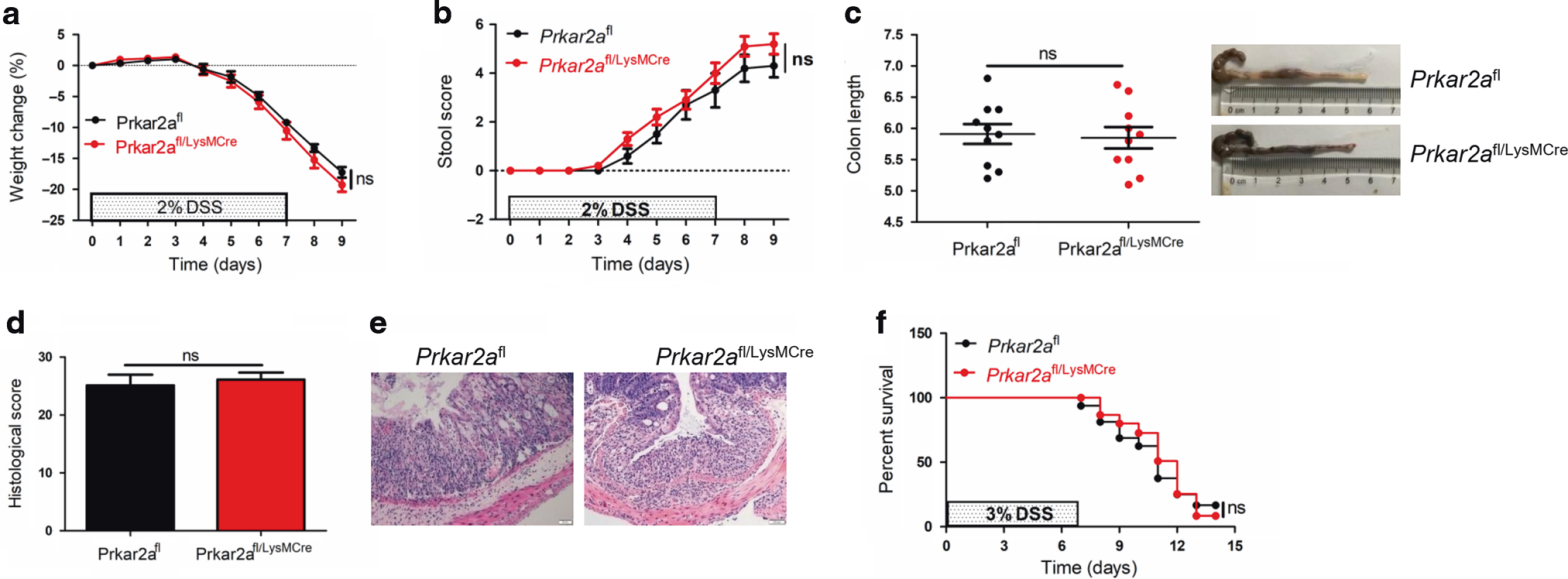
Ablation of Prkar2a in immune cells does not protect mice against DSS-induced colitis. **a**–**e** *Prkar2a*^fl^ (*n* = 10) and *Prkar2a*^fl/LysMCre^ (*n* = 10) mice were treated with 2% DSS for 1 week followed by 2 days of normal drinking water. **a** Weight loss was monitored daily and is displayed as the percentage of the initial body weight. **b** Stool score was measured every day during colitis development. **c** At day 9, colons were removed and colon lengths were determined. **d** Histological analysis of distal colon tissues at day 9 of experimental colitis. **e** Representative images of distal colon at day 9. Scale bar = 50 μm. **f** The survival curve of *Prkar2a*^fl^ (*n* = 14) and *Prkar2a*^fl/LysMCre^ (*n* = 14) mice. Mice were treated with 3% DSS for 1 week and the mortality was monitored over 14 days. Log-rank (Mantel–Cox) test was used to do the analysis. Data shown in **a**–**f** are representative of two independent experiments. All graphs show mean ± SEM. Student’s *t* test (**c**, **d**) or two-way ANOVA (**a**, **b**) was used to compare experimental groups. ns not significant.

To address the role of PRKAR2A in myeloid cells, we crossed LysM-Cre mice^21^ with *Prkar2a*^fl^ mice and generated mice that specifically lack *Prkar2a* in the myeloid lineage. As shown in Fig.4, *Prkar2a*^fl/LysMCre^ mice exhibited comparable weight loss (Fig. 4a), stool score (Fig. 4b), colon length (Fig. 4c), and histological score (Fig. 4d, e) as compared to *Prkar2a*^fl^ mice. Additionally, *Prkar2a*^fl/LysMCre^ mice and *Prkar2a*^fl^ mice exhibited similar mortality after 3% DSS administration (Fig. 4f). Taken together, our results suggest that *Prkar2a* ablation in myeloid cells does not protect the mice against DSS-induced colitis.

### PRKAR2A deficiency influences colonic epithelial homeostasis in DSS-induced colitis

**Fig. 5:**
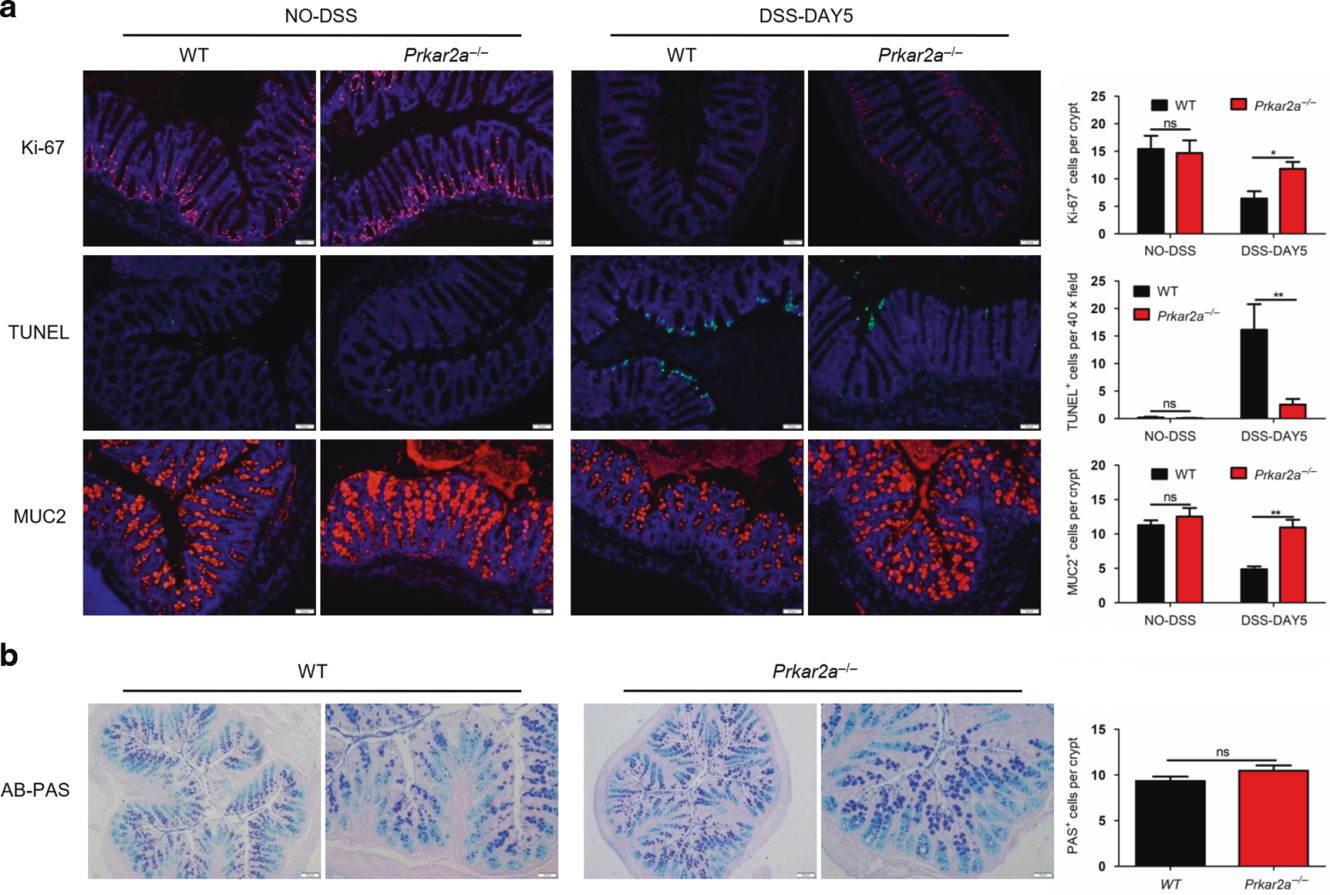
*Prkar2a*^−/−^ mice exhibit altered colonic epithelial homeostasis during DSS-induced colitis. **a** Immunofluorescent stain of Ki-67, TUNEL, and goblet cell marker MUC2 in colon tissues of WT (*n* = 3) and *Prkar2a*^−/−^ mice (*n* = 3) treated or untreated with DSS. Quantification of Ki-67-, TUNEL-, and MUC2-positive epithelial cells is shown in the right panel. Scale bar = 50 µm. Data shown in **a** are representative of at least three independent experiments. **b** Representative images of Alcian Blue-PAS (AB-PAS) staining of colon tissues of WT (*n* = 3) and *Prkar2a*^−/−^ mice (*n* = 3), and quantification of PAS+IECs per crypt in AB-PAS-stained colon sections is shown in the right panel. Scale bar (left panel of each group) = 100 µm. Scale bar (right panel of each group) = 50 µm. Five images per mouse were quantified. Data shown in **b** are representative of two independent experiments. All data are mean ± SEM. Student’s *t* test was used to compare experimental groups. **P < 0.01; *P < 0.05. ns not significant.

Colonic epithelial homeostasis plays a vital role in the development of colitis. To investigate whether the lack of PRKAR2A influences epithelial turnover, we analyzed the proliferation and apoptosis of colonic epithelial cells and goblet cell number before and after DSS treatment. At the steady state, *Prkar2a*^−*/*−^ mice and WT littermates showed no significant differences in colonic epithelial proliferation and apoptosis (Fig. 5a). However, after DSS treatment, *Prkar2a*^−*/*−^ mice exhibited a significantly lower decrease in proliferation, as indicated by the increased Ki-67^+^ IECs per crypt, when compared with those of WT controls (Fig. 5a). Terminal deoxynucleotidyl transferase-mediated dUTP-fluorescein nick end labeling (TUNEL) staining demonstrated a lower number of apoptotic IECs in *Prkar2a*^−*/*−^ mice than in WT mice (Fig. 5a). Goblet cell depletion has been regarded as an important characteristic associated with mucosal inflammation^22^. Therefore, we determined whether PRKAR2A deficiency could influence goblet cell number. The percentage of MUC2^+^ goblet cells was comparable in WT and *Prkar2a*^−*/*−^ mice at the steady state, while during DSS-induced colitis, the number of MUC2^+^ goblet cells in *Prkar2a*^−*/*−^ mice was higher than that in WT mice, suggesting a lower goblet cell loss in *Prkar2a*^−*/*−^ mice during colon inflammation (Fig. 5a). We further confirmed goblet cell number via Alcian Blue–Periodic acid-Schiff (AB-PAS) staining and found that PRKAR2A deficiency did not affect goblet cell number at steady state (Fig. 5b).

Taken together, our data indicate that, after DSS treatment, *Prkar2a*^−*/*−^ mice exhibited lower decrease in proliferation, less apoptosis of colon epithelium, and less goblet cell loss as compared to WT mice. These results are consistent with the amelioration of colitis in *Prkar2a*^−*/*−^ mice. However, PRKAR2A deficiency did not influence colonic epithelial homeostasis at the steady state.

### PRKAR2A modulates signal transducer and activator of transcription factor 3 (STAT3) activation in an interleukin (IL)-6-independent manner

**Fig. 6:**
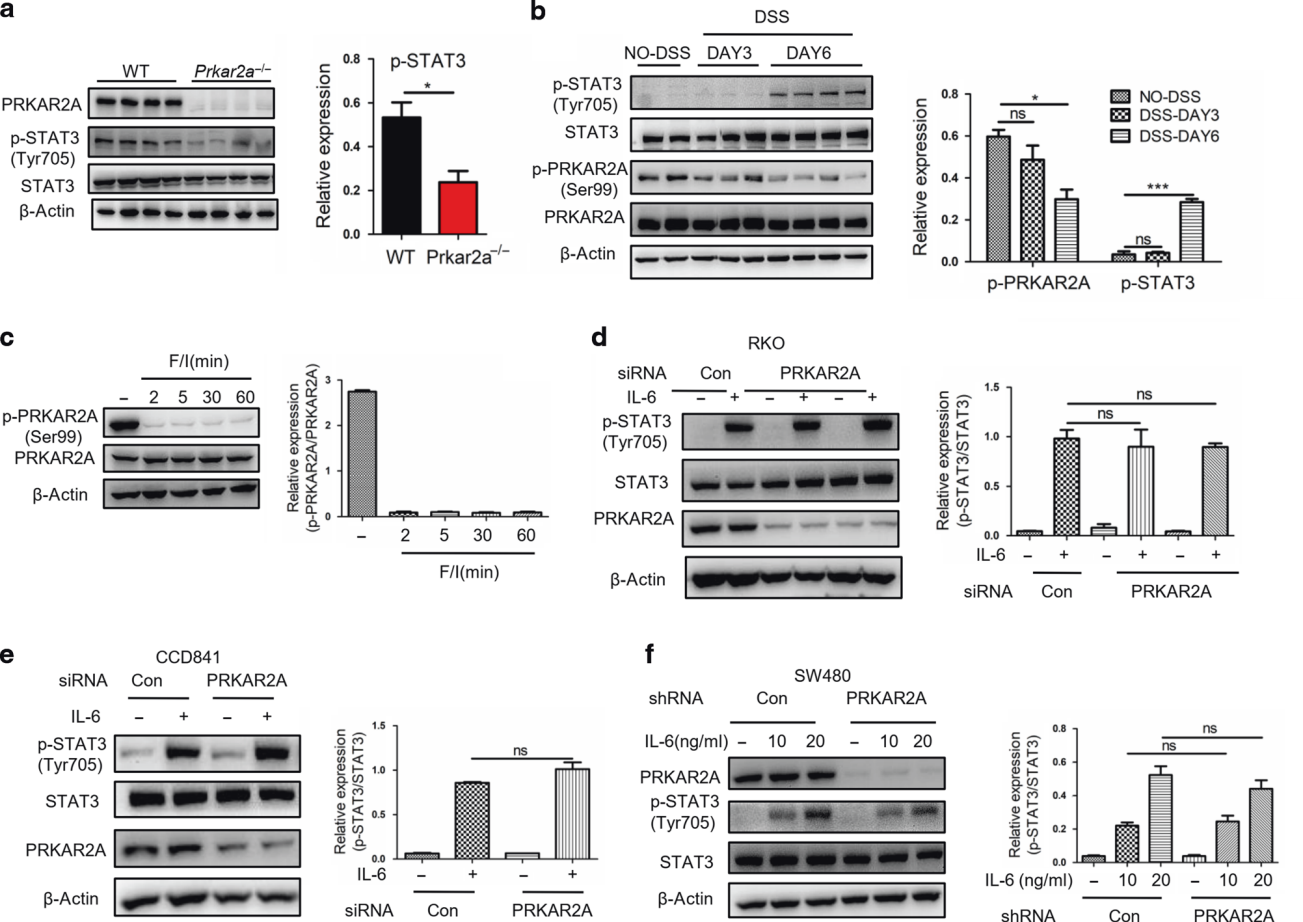
*Prkar2a* deficiency inhibits STAT3 activation through an IL-6-independent pathway. **a** Western blot analysis of STAT3 activation in colon tissues collected from *Prkar2a*^−/−^ (*n* = 4) and WT mice (*n* = 4) at day 6 of DSS-induced colitis. Data are representative of at least three independent experiments. **b** Immunoblot analysis of the indicated proteins in colon tissues from WT mice at day 0 (*n* = 2), day 3 (*n* = 3), and day 6 (*n* = 4) of DSS-induced colitis. Data are representative of two independent experiments. **c** Western blot analysis of the indicated proteins in CCD841 cell line after stimulation with F/I. Data are representative of at least three independent experiments. **d** Immunoblot analysis of the indicated proteins in RKO cells transfected with siRNA against PRKAR2A or a scramble control and then stimulated with recombinant human IL-6 (10 ng/mL) for 30 min. **e** Immunoblot analysis of the indicated proteins in CCD841 cells transfected with siRNA against PRKAR2A or a scramble control and then stimulated with recombinant human IL-6 (10 ng/mL) for 30 min. **f** Immunoblot analysis of the indicated proteins in SW480 cells after stable knockdown of PRKAR2A followed by stimulation with recombinant human IL-6 for 30 min. Data shown in **d**–**f** are representative of two independent experiments conducted in duplicate. Student’s *t* test (**a**, **e**, **f**) or two-way ANOVA (**b**) or one-way ANOVA (**d**) was used to do the analysis. ***P < 0.001; *P < 0.05. ns not significant.

STAT3 is a transcription factor that is activated by a variety of cytokines and growth factors. An excessive accumulation of activated STAT3 in colonic epithelial cells and lamina propria mononuclear cells has been observed in patients with UC and DSS-induced experimental colitis^23–25^. Decreased STAT3 activation was observed in the colon tissues of *Prkar2a*^−/−^ mice than in WT mice after DSS administration (Fig. 6a). Furthermore, we found that STAT3 was activated at a late time point in experimental colitis, that is, on day 6 post DSS treatment (Fig. 6b), which was in line with a previous study^24^. However, PKA was activated at an early time point, as demonstrated by p-PRKAR2A, which was downregulated on day 3 of colitis (Fig. 6b). It has been reported that PRKAR2A autophosphorylation at Ser99 occurs in the inactive PKA holoenzyme and that it disappears on PKA activation^26^. We confirmed an obvious dephosphorylation of P-Ser99 on PRKAR2A in the colonic epithelial cell line CCD841 through PKA activation by Forskolin and IBMX (Fig. 6c). This phenomenon allowed us to directly monitor the activity of PKA using western blotting and immunohistochemistry of p-PRKAR2A(ser99), which indicates inactive PKA, and the decrease of p-PRKAR2A(ser99) as an indication of PKA activation. As STAT3 activation was suppressed in *Prkar2a*^−/−^ mice and PKA was activated before STAT3 during colitis, we hypothesized that PRKAR2A might influence experimental colitis by modulating STAT3 signaling.

IL-6-induced STAT3 signaling has been demonstrated to play an active role in regulating the inflammatory processes in patients with IBD and experimental colitis^25,27^. To determine whether PRKAR2A regulates STAT3 activation through IL-6, we transiently transfected human colorectal cancer cell line RKO with specific PRKAR2A small interfering RNA (siRNA) or control siRNA, and a clear knockdown of PRKAR2A was achieved (Fig. 6d). However, PRKAR2A silencing had little impact on IL-6-induced STAT3 activation, as demonstrated by a lack of significant changes in p-STAT3 after stimulation with IL-6 (10 ng/mL) in PRKAR2A knockdown cells as compared to that in control cells (Fig. 6d). The same result was observed in the human normal epithelial cell line CCD841 (Fig. 6e). To confirm these results, we stably knocked down PRKAR2A through lentiviral transduction of short hairpin RNA (shRNA) in the human colorectal cancer cell line SW480. Consistent with transient knockdown experiments, a stable silencing of PRKAR2A had no impact on IL-6-induced STAT3 activation, even when 20 ng/mL IL-6 was used. Collectively, our data suggest that PKA activation is an early event in experimental colitis, and the disassociated PRKAR2A after PKA activation might take part in the subsequent STAT3 activation, but it is not dependent on the classical IL-6 signaling.

### *Prkar2a* deficiency upregulates type I IFN-induced ISGs and inhibits type I IFN-induced STAT3 activation

**Fig. 7:**
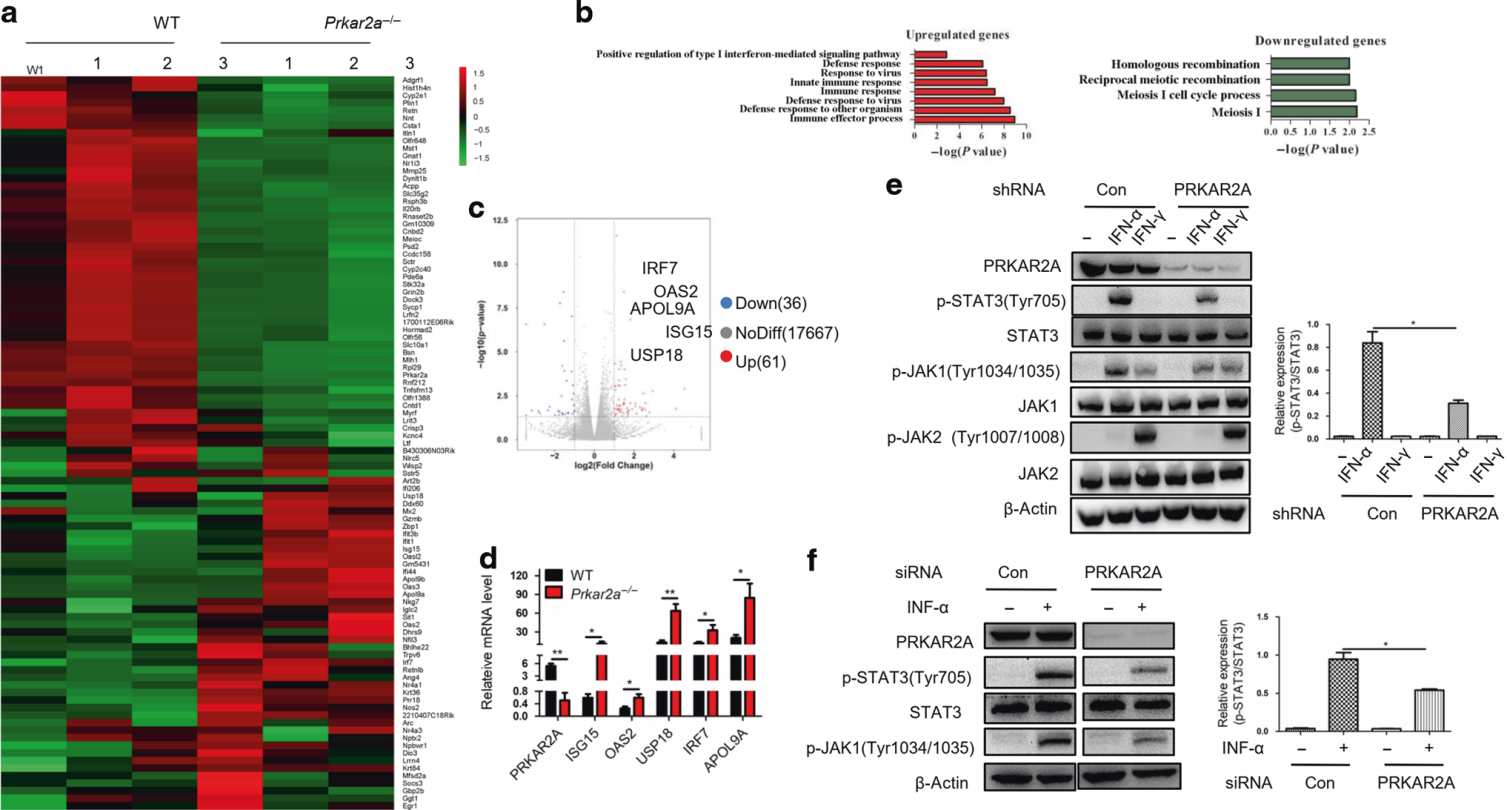
PRKAR2A orchestrates type I IFN-induced signaling pathway. **a**–**c** Colon tissues from WT (*n* = 3) and *Prkar2a*^−/−^ mice (*n* = 3) were subjected to RNA-seq. **a** Heatmap of RNA-seq data shows at least twofold upregulated or downregulated expression of genes in colon tissues from WT and *Prkar2a*^−/−^ mice. **b** GO analysis of the upregulated and downregulated genes in colon tissues in *Prkar2a*^−/−^ mice. **c** Volcano plot of the RNA-seq data. **d** Quantitative PCR analysis of PRKAR2A, OAS2, APOL9A, ISG15, IRF7, and USP18 mRNA levels in colon tissues from *Prkar2a*^−/−^ (*n* = 3) and WT mice (*n* = 3). **e** PRKAR2A expression was stably knocked down in SW480 cells. The cells were then stimulated with IFN-α (200 ng/mL) or IFN-γ (50 ng/mL) for 30 min, and the indicated proteins were detected by western blot. **f** Expression of PRKAR2A was knocked down by siRNA in CCD841 cells. The cells were stimulated with or without IFN-α (200 ng/mL) for 30 min, and the indicated proteins were detected by western blot. Data shown in **d**–**f** are representative of three independent experiments conducted in duplicate. Error bars represent mean ± SEM. Student’s *t* test was used to compare the experimental groups. *P < 0.05, **P < 0.01.

To elucidate the potential pathways involved in the protection against DSS-induced colitis after PRKAR2A ablation, we performed whole-transcriptome analysis of colon tissues from WT and *Prkar2a*^−*/*−^ mice using RNA sequencing (RNA-seq). The heatmap displayed distinct gene expression profiles between *Prkar2a*^−*/*−^ and WT mice (Fig. 7a). Gene ontology analysis highlighted that PRKAR2A deficiency upregulated the genes that positively regulate type I IFN-mediated signaling pathway and downregulated the genes associated with meiosis and homologous recombination (Fig. 7b). The expression levels of 61 genes out of the 17764 examined expression tags were upregulated more than twofold, whereas the expression of 36 genes decreased more than twofold in *Prkar2a*^−/−^ mice than in WT mice (Fig. 7c). Additionally, a significant upregulation of type I IFN-induced ISGs (IRF7, OAS2, APOL9A, USP18, and ISG15) expression was observed in *Prkar2a*^−*/*−^ mice (Fig. 7a, c). Quantitative PCR was performed to confirm RNA-seq results. In line with the RNA-seq data, the expression of ISGs was significantly increased in the colon of *Prkar2a*^−*/*−^ mice than in the colon of WT controls (Fig. 7d). Our data demonstrated that PRKAR2A ablation activates the classical type I IFN signaling pathway in colon tissues.

Type I IFN, mainly IFN-α and IFN-β, has been reported to induce STAT3 activation in various cell types^28^. Our results prompted us to determine whether the decreased p-STAT3 in inflamed colon tissues of *Prkar2a*^−/−^ mice was due to an altered type I IFN signaling. After stable knockdown of PRKAR2A by shRNA in SW480 cells, we found that the IFN-α-induced activation of STAT3 was obviously downregulated, whereas PRKAR2A deficiency had no impact on the type II IFN (IFN-γ) signaling pathway (Fig. 7e). To confirm this, we silenced PRKAR2A in CCD841 cells and stimulated the cells with IFN-α. Similar with the observation in SW480 cells, PRKAR2A ablation significantly suppressed IFN-α-induced STAT3 activation (Fig. 7f). These data suggest that the inhibited STAT3 activation in *Prkar2a*^−/−^ mice is dependent on type I IFN signaling.

It is interesting that the level of p-CREB (ser133) and CBP were increased in *Prkar2a*^−/−^ mice than in WT controls (Fig. S1a, b). As p-CREB is a well-known downstream signaling of PKA catalytic subunit, we wanted to know whether PKA catalytic subunit participates in regulating type I IFN-induced STAT3 activation. We knocked down PRKACA, a widely expressed PKA catalytic subunit, in RKO and CCD841 cells. However, PRKACA silencing had little impact on type I IFN-induced STAT3 activation (Fig. S1c, d). Our data suggest that the decreased type I IFN-induced STAT3 activation in *Prkar2a*^−/−^ mice has nothing to do with PKA catalytic subunit.

### *Prkar2a* deficiency ameliorates experimental colitis in part through ISGs

**Fig. 8:**
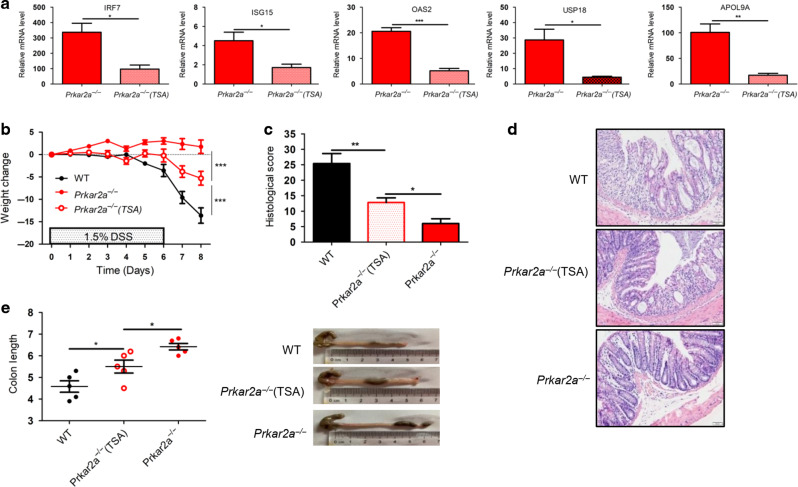
Inhibiting ISGs partly reverses the protection against DSS-colitis in *Prkar2a*^−/−^ mice. **a** RT-qPCR analysis ISGs (IRF7, ISG15, OAS2, USP18, and APOL9A) in colon tissues of *Prkar2a*^−/−^ mice treated (*n* = 3) or untreated (*n* = 3) with TSA. Data are representative of two independent experiments conducted in duplicate. **b**–**e** *Prkar2a*^−/−^ mice (*n* = 5), TSA-treated *Prkar2a*^−/−^ mice (*n* = 5), and WT mice (*n* = 5) were challenged with 1.5% DSS for 6 days followed by 2 days of normal water to induce experimental colitis. **b** Weight loss was determined daily and is displayed relative to initial weight. **c** Histological scores. **d** Representative H&E-stained images of colon sections. Scale bar = 50 µm. **e** At day 9, the mice were sacrificed and colons were removed to calculate the length. Data shown in **b**–**e** are representative of three independent experiments. Results are presented as mean ± SEM. Student’s *t* test (**a**) or one-way ANOVA (**c**, **e**) or two-way ANOVA (**b**) was used to compare the experimental groups. *P < 0.05, **P < 0.01, ***P < 0.001.

Classically, type I IFN-induced ISGs are potent antiviral immune regulators. However, many studies have demonstrated that type I IFN plays an important role in maintaining intestinal homeostasis, and modulating type I IFN signaling pathway may be of therapeutic value in intestinal inflammatory conditions^29–31^. To assess the possible contribution of ISGs to the amelioration of DSS-induced colitis in *Prkar2a*^−/−^ mice, we used trichostatin A (TSA) to inhibit ISGs. TSA is a potent general inhibitor of class I and II histone deacetylase enzymes, which are required for the transcriptional activation of ISGF3-responsive genes^32,33^. TSA was administered intraperitoneally (i.p.) to *Prkar2a*^−/−^ mice daily for 2 weeks to inhibit ISG expression in vivo. As expected, TSA efficiently inhibited the expression of ISGs in colon tissues (Fig. 8a). After challenging with DSS, TSA-treated *Prkar2a*^−/−^ mice showed exacerbated colitis as compared to non-TSA-treated *Prkar2a*^−/−^ mice, as assessed by an accelerated weight loss (Fig. 8b), higher histological score (Fig. 8c–d), and severe colon shortening (Fig. 8e). However, the severity of colitis in TSA-treated *Prkar2a*^−/−^ mice was still milder than that in WT mice (Fig. 8b–e), suggesting that ISGs inhibition cannot completely reverse the resistance to DSS-induced colitis in *Prkar2a*^−/−^ mice. In summary, our results indicated that the amelioration of colitis in *Prkar2a*^−/−^ mice was partly due to elevated ISGs, and other unknown mechanisms might exist that contribute to the protection against colitis in *Prkar2a*^−/−^ mice.

### *Prkar2a* deficiency alters the gut microbial composition

**Fig. 9:**
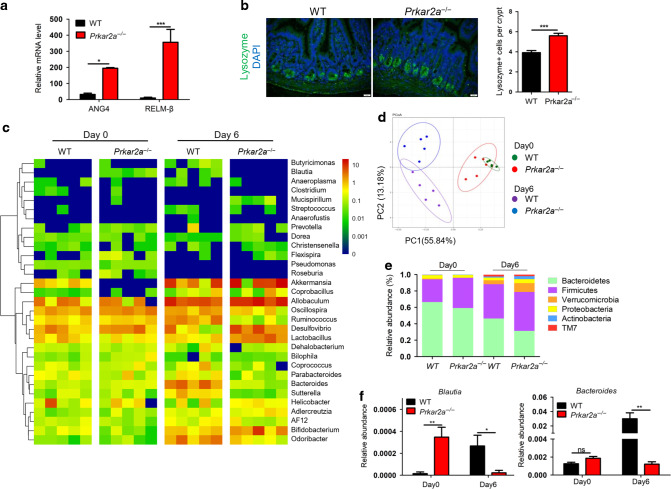
Ablation of Prkar2a alters gut bacterial composition. **a** RT-qRCR analysis of the transcription level of ANG4 and RELMβ in colon tissues collected from *Prkar2a*^−/−^ (*n* = 3) and WT (*n* = 3) mice. Data are representative of three independent experiments conducted in duplicate. **b** Lysozyme immunostaining shows the increased Paneth cells in *Prkar2a*^−/−^ mice (*n* = 3) and WT mice (*n* = 3). Quantification of Lysozyme-positive cells per crypt. Five images per mouse were quantified. Data are representative of two independent experiments. **c** Heatmap depicting of relative abundance of microbiota at the family level in colon contents collected from *Prkar2a*^−/−^ and WT mice at day 0 or day 6 of DSS-induced colitis (*n* = 5 per group). **d** Principal coordinate analysis (PCoA) of weighted UniFrac distances based on 16S rDNA analysis of microbiota of colon contents from *Prkar2a*^−/−^ and WT mice pre- and post-DSS challenge (*n* = 5 per group). Symbols represent data from individual mice. **e** Relative abundances of microbial commensal diversity were analyzed at the phylum level by 16S rRNA gene sequencing. **f** Relative abundances of Blautia genera and Bacteroides genera in *Prkar2a*^−/−^ mice versus WT mice before and after DSS challenge. Data are presented as mean ± SEM. Student’s *t* test was used to do the analysis. *P < 0.05, **P < 0.01, ***P < 0.001, ns not significant.

Gut microflora is regarded as one of the major factors determining the sensitivity to colitis. Our previous RNA-seq data showed that *Prkar2a*^−/−^ mice possess elevated transcript levels of antimicrobial proteins, RELMβ and ANG4, in colon tissues, and this was further confirmed using reverse transcription PCR (Fig. 9a). Moreover, the increased antimicrobial proteins might due to the increased numbers of Paneth cells, as *Prkar2a*^−/−^ mice exhibited more Lysozyme-positive cells compared with WT controls (Fig. 9b). Antimicrobial proteins can regulate the composition of intestinal microbial communities. To evaluate whether PRKAR2A deficiency influences the gut microbial ecosystem, 16S rRNA gene sequencing was used to comprehensively analyze the bacterial composition in the colon of *Prkar2a*^−/−^ and WT mice. The heatmap showed that the relative abundance of fecal bacteria composition was different between *Prkar2a*^−/−^ mice and WT controls (Fig. 9c). Further, the principal coordinate analysis of weighted UniFrac distances revealed a separation between the genotypes at steady state, which was more evident after DSS challenge (Fig. 9d). At the phylum level, a greater abundance of *Firmicutes* and a lower abundance of *Bacteroides* were observed in *Prkar2a*^−/−^ mice than in WT controls, regardless of DSS treatment (Fig. 9e). Moreover, *Prkar2a*^−/−^ mice exhibited less abundance of the genera *Bacteroides* and *Blautia*, as compared to WT controls during DSS-induced colitis development. However, the abundance of *Blautia* in *Prkar2a*^−/−^ mice was higher than that in WT controls at the steady state (Fig. 9f). Taken together, our data suggest that PRKAR2A deficiency alters the gut microbiota.

### Resistance to DSS-induced colitis in *Prkar2a*^−/−^ mice is largely dependent on the gut microbiota

**Fig. 10:**
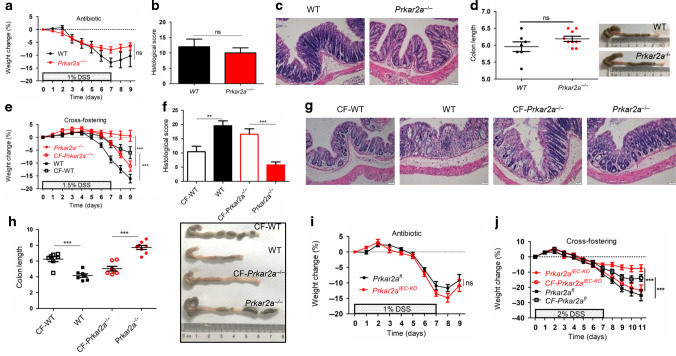
Intestinal microbiota contributes to the ameliorated colitis in *Prkar2a*-dificiency mice. **a**–**d** After treated with broad-spectrum antibiotics for 4 weeks, WT (*n* = 7) and *Prkar2a*^−/−^ mice (*n* = 9) were given 1% DSS water for 7 days, followed by 2 days of regular water. Mice were sacrificed at day 9. Data shown in **a**–**d** are representative of three independent experiments. **a** Body weight change. **b** Histological scores. **c** Representative H&E-stained images of colon sections. Scale bar = 50 μm. **d** Graphical presentation of the colon length and photographs of colons. **e**–**h** Newborn WT and Prkar2a^−/−^ mice were cross-fostered with *Prkar2a*^−/−^ and WT mothers, respectively (*n* = 7–8). Eight weeks later, 1.5% DSS water were given for 7 days to induce colitis, followed by 2 days normal water. The mice were sacrificed on day 9. Data shown in **e**–**h** are representative of two independent experiments. **e** Body weight change. **f** Histological scores. **g** Representative H&E-stained images of colon sections. Scale bar = 50 μm. **h** Photographs of colons and graphical presentation of the colon length. **i** After treated with broad-spectrum antibiotics for 4 weeks, *Prkar2a*^fl^ (*n* = 6) and *Prkar2a*^IEC-KO^ (*n* = 7) mice were given 1% DSS water for 7 days to induce colitis, followed by 2 days of normal water. Body weight was monitored daily over a period of 9 days. Data shown in **i** are representative of two independent experiments. **j** Newborn *Prkar2a*^fl^ and *Prkar2a*^IEC-KO^ mice were cross-fostered with *Prkar2a*^IEC-KO^ and *Prkar2a*^fl^ mothers, respectively (*n* = 7–14). After 8 weeks, 2% DSS water were given for 7 days to induce colitis. Body weight was monitored daily. Data shown in **j** are representative of two independent experiments. Data are presented as mean ± SEM. Student’s *t* test (**b**, **d**, **f**, **h**) or two-way ANOVA (**a**, **e**, **i**, **j**) was used to do the analysis. **P < 0.01, ***P < 0.001, ns not significant.

To verify whether an altered microbiota contributes to the ameliorated colitis in *Prkar2a*^−/−^ mice, we treated WT and *Prkar2a*^−/−^ mice with drinking water containing a broad-spectrum antibiotic cocktail for 4 weeks to eliminate the gut luminal bacteria before DSS administration. After treatment with broad-spectrum antibiotics, *Prkar2a* deficiency failed to protect mice from DSS-induced colitis, as indicated by comparable weight loss (Fig. 10a), histological score (Fig. 10b, c), and colon length (Fig. 10d) in WT and *Prkar2a*^−/−^ mice. It has been reported that an abnormal gut microflora is transmissible and correlated with the sensitivity to colitis. To further establish the role of intestinal microbiota, we performed cross-fostering experiments. *Prkar2a*^−/−^ mice were cross-fostered (CF) with WT mothers at birth (CF-*Prkar2a*^−/−^), and they exhibited severe colitis as compared to non-CF *Prkar2a*^−/−^ mice (Fig. 10e–h). In contrast, newborn WT mice cross-fostered with *Prkar2a*^−/−^ mothers (CF-WT) developed milder colitis as compared to non-CF WT mice (Fig. 10e–h). Additionally, the protection against experimental colitis in *Prkar2a*^IEC−KO^ mice also disappeared after treatment with broad-spectrum antibiotics (Fig. 10i). The cross-fostering experiments further confirmed that the ameliorated state of colitis in *Prkar2a*^IEC−KO^ mice was largely due to the intestinal microbiota (Fig. 10j). Collectively, our data demonstrate that an altered gut microbiota is the primary cause of a lower sensitivity to DSS-induced colitis in *Prkar2a*^−/−^ mice.

## Discussion

In this study, for the first time, we reported that PRKAR2A deficiency protects mice against DSS-induced experimental colitis by facilitating ISG expression and modulating the gut microbiota.

Interestingly, although *Prkar2a*^−/−^ mice exhibited an ameliorated DSS-induced colitis, STAT3 activation decreased in *Prkar2a*^−/−^ mice than in WT mice during acute colitis. This seems inconsistent with previous studies that reported that STAT3 activation in IECs protects mice from experimental colitis^24^ and loss of intestinal IL-6-STAT3 signaling exacerbates acute colitis^34–36^. However, in our experiments, the reduced STAT3 activation in *Prkar2a*^−/−^ mice was not dependent on classical IL-6 signaling, as demonstrated by no impact on IL-6-induced STAT3 activation after PRKAR2A silencing ex vivo. Through RNA-seq analysis, we found an unexpected relationship between PRKAR2A and type I IFN. In addition to IL-6, type I IFN and type II IFN can also activate STAT3. Furthermore, we confirmed that silencing PRKAR2A inhibited type I IFN-induced STAT3 activation, suggesting that the suppression of STAT3 activation in *Prkar2a*^−/−^ mice might be dependent on type I IFN. Although STAT3 can be activated by many inflammatory factors such as IL-6, IL-22, tumor necrosis factor-α, and IFN, its downstream effectors are different. It has been reported that IFNα stimulation failed to activate the transcription of well-established target genes of the STAT3 signaling pathway, such as *SOCS3* and *c-FOS*^37^. Based on our experiments, we presumed that, unlike classical IL-6-induced STAT3 activation, type I IFN-induced STAT3 might not play a protective role in DSS-induced colitis. However, little is known about the effects of type I IFN-induced STAT3 activation in colonic inflammation. Several studies have reported that STAT3 mediates the suppression of IFN antiviral responses in some cell types, and STAT3 inhibition amplifies the induction of ISGs^38–40^. Additionally, previous studies have proposed that type I IFN triggers the following competitive pathways: (1) a STAT1-dependent dominant signaling cascade that accounts for the principal type I IFN biological properties, (2) a STAT3-dependent non-classical signaling cascade, which exhibit an inhibitory effect on the dominant STAT1-ISG pathway^41^. We found that PRKAR2A deficiency increased type I ISG expression and suppressed type I IFN-induced STAT3 activation. Thus, we speculated that PRKAR2A ablation promoted ISG expression via inhibiting type I IFN-induced STAT3 activation. Although type I IFN is typically considered to be the most important in viral responses, several studies have reported that type I IFN and downstream ISGs play an important role in host mucosal defense and attenuation of experimental colitis^42–44^. These studies are in line with our findings that type I IFN-induced ISGs contributed to the protective effect in *Prkar2a*^−/−^ mice in experimental colitis.

The gut microbiota exerts a great influence on host health and diseases, and the microbiota composition is critical for maintaining intestinal homeostasis. In the present study, we discovered that PRKAR2A deficiency altered the colon microbiota composition in mice. Most notably, *Prkar2a*^−/−^ mice exhibited a reduced abundance of *Bacteroides* and *Blautia* after DSS treatment. *Bacteroides* have specific colitogenic effects that can induce colitis in susceptible animals^45^. The species *Ruminococcus gnavus*, which is classified as a member of the *Blautia* genus, has been reported to be enriched in IBD patients^46^. These researches suggest that the reduced abundance of *Bacteroides* and *Blautia* in *Prkar2a*^−/−^ mice after DSS treatment might contribute to the amelioration of colitis. Interestingly, although the abundance of *Blautia* in *Prkar2a*^−/−^ mice decreased during colitis, the relative abundance of *Blautia* was higher in *Prkar2a*^−/−^ mice than in WT mice at the steady state. Further investigations of the *Blautia* are needed to better understand the relationship between gut bacteria and IBD. Indeed, little difference was observed in the severity of DSS-induced colitis between WT and *Prkar2a*^−/−^ mice after eliminating the gut bacteria with antibiotics. Cross-fostering can induce a permanent microbiota shift, which is shaped by the nursing mother, and it is an effective means to evaluate the influence of commensal microbiota on colitis^47^. Our cross-fostering experiments further confirmed that gut microbiota primarily contributed to the ameliorated colitis in *Prkar2a*^−/−^ mice. However, the genus or species that mainly contributes to this protection requires further investigation.

Our work highlights another side of PKA signaling in colonic inflammation. In the classical PKA signaling pathway, PKA catalytic subunits are considered to be responsible for signal transduction. However, little is known about the PKA regulatory subunits. Although many studies suggest that the activation of PKA signaling has a powerful anti-inflammatory effect^13^, a clinical trial of a PDE4 inhibitor has shown unsatisfactory result^14^. Our findings suggest that PRKAR2A might contribute to the unsatisfactory results of PDE4 inhibitor clinical trial, as the disassociated PRKAR2A after PKA activation might exacerbate intestinal inflammation. Hence, it is possible that PKA activation combined with PRKAR2A inhibition might be effective in achieving a better anti-inflammatory effect of IBD treatment.

In this study, we report a previously unidentified function of PRKAR2A deficiency in ameliorating DSS-induced colitis. Our results provide a link between PRKAR2A, type I IFN, gut microbiota, and intestinal inflammation (Fig. S2), suggesting that PRKAR2A inhibition might be a potential therapeutic strategy in treatment for human IBD.

## Methods

### Mice

*Prkar2a*^−/−^ mice and *Prkar2a*^fl^ mice were kindly provided by Professor Ying Yu (Shanghai Institute of Nutrition and Health, CAS); Villin-Cre mice and LysM-Cre mice were purchased from Model Animal Research Center of Nanjing University. *Prkar2a*^fl^ mice were crossed with Villin-Cre or LysM-Cre mice to obtain *Pkrar2a*^IEC−KO^ or *Prkar2a*^fl/LysM^ mice. Genotyping was performed on the tail DNA of 4-week-old pups as described in our previous work^18,48^. All mice were on the C57BL/6 background, 5–6 mice every cage, had water ad libitum, and were fed regular chow. All mice were kept at a constant temperature (23 ± 1 °C) and humidity (40 ± 10%) under a strict 12-h light cycle (lights on at 7:00 a.m. and off at 7:00 p.m.). All caging, bedding, and food were sterilized prior to use, and all mice experiments were performed in a biosafety cabinet. If not stated otherwise, mice were kept separated by genotype and gender. All experiments were performed with gender- and age-matched controls. Animals were housed in individual ventilated caging system under specific pathogen-free conditions at the animal facility of Shanghai Institute of Nutrition and Health, Chinese Academy of Sciences, and all animal procedures were approved by the Institutional Animal Care and Use Committee of the Shanghai Institute of Nutrition and Health, Chinese Academy of Sciences.

### Human samples

Human samples from IBD patients and uninflamed controls were approved by the Ethics Committee of Ruijin Hospital Affiliated to Shanghai Jiao Tong University School of Medicine, and informed consent was obtained from all subjects.

### Induction and assessment of experimental colitis

Mice aged 8–10 weeks of matched genders were given 1–3% (w/v) DSS (MP Biomedicals) in drinking water for 6 or 7 days to induce experimental colitis. Where indicated, mice were injected i.p. with 1 mg/kg TSA (Sigma-Aldrich) or 0.9% NaCl as control for 2 weeks before challenged with DSS. Body weight changes were calculated as a percentage relative to the weight prior to DSS treatment. Stool score was determined based on stool consistency and rectal bleeding as described previously^49^. Briefly, the scores were calculated as follows: stool blood (0, negative; 2, positive; 4, gross bleeding), and stool consistency (0: formed and hard, 1: formed but soft, 2: loose stools, 3: mid diarrhea, watery, 4: diarrhea). Stool blood was tested using Hemoccult cards (Beckman Coulter). Upon sacrifice, large intestines were removed and flushed with phosphate-buffered saline (PBS). Distal colon pieces were either frozen in liquid N_2_ for RNA or protein isolation or fixed in 4% paraformaldehyde. H&E-stained paraffin sections were imaged and used for histopathological evaluation. Histological score was calculated as described previously^50^. Briefly, it included three criteria: the severity of inflammation (0–3), the level of involvement (0–3), and the extent of epithelial/crypt damage (0–4). Each parameter was then multiplied by a factor reflecting the percentage of the colon involved (0–25%, 26–50%, 51–75%, and 76–100%) and then summed to obtain the overall score.

### Antibiotic experiment

For antibiotic treatment, the drinking water was replaced with filter-sterilized water containing ampicillin (1 g/L; Sigma-Aldrich), vancomycin (0.5 g/L; Sigma-Aldrich), neomycin (1 g/L; Sigma-Aldrich), and metronidazole (1 g/L; Sigma-Aldrich). Antibiotic-containing water was replaced at least once a week during the course of the experiment.

### Cross-fostering

Breeding pairs of WT and *Prkar2a*^−/−^ mice (or *Prkar2a*^fl^ and *Prkar2a*^IEC−KO^ mice) were simultaneously set up when individual mice reached approximately 8 weeks of age. Fourteen days after introduction, females were monitored daily for pregnancy stage and the males were removed from pregnant females. Newborn mice were exchanged within 24 h of birth. After the birth of both WT and *Prkar2a*^−/−^ mice (or *Prkar2a*^fl^ and *Prkar2a*^IEC−KO^ mice), several cages of pups were exchanged to the mother of the opposite strain, and several cages of pups were not exchanged and remained with the birth mother. The pups were nursed by their respective mothers until weaning (postnatal day 21). At weaning, pups were separated based on sex, strain, and nursing mother.

### Isolation of IECs and flow cytometry

Mouse IECs were isolated as previously described^51^. Colons were isolated, cut into 2–3-mm pieces, and rinsed in cold PBS. Tissue pieces were then shaken at 37 °C in Hank’s Balanced Salt Solution containing 5 mM EDTA and 1 mM dithiothreitol for 20 min. The supernatant was centrifuged (1000 × *g*, 5 min) and washed three times in cold PBS, and cell pellets were then lysed in RIPA buffer and total proteins were extracted. For flow cytometric analysis, isolated single epithelial cell suspensions were stained by standard protocol with the following antibodies: PerCP-Cy5.5–conjugated CD45 (BD Biosciences) and APC–conjugated Ep-CAM (BioLegend). Flow cytometry was performed using a FACSCalibur flow cytometer (BD Biosciences).

### Immunofluorescence

For whole-tissue immunofluorescence, human samples or colons harvested from mice were frozen in Tissue-Tek OCT compound, cryosectioned by using Leica CM3050 S cryostats, fixed in acetone, washed, and blocked with PBS containing 5% goat serum. The sections were incubated at 4 °C overnight with primary antibodies to detect PRKAR2A (1:100; Santa Cruz Biotechnology), p-PRKAR2A(ser99) (1:100, Santa Cruz Biotechnology), Pankeratin (1:100; Cell Signaling), Ki-67 (1:200; Abcam), and MUC2 (1:200; Abcam). The sections were then washed, incubated with the corresponding secondary antibodies labeled with Alexa Fluor 488 or 594 (1:1000, Invitrogen) for 1 h, washed again, and mounted onto coverslips by using mounting solution with DAPI (Prolong Gold). Apoptosis was analyzed using DeadEnd™ Fluorometric TUNEL System (Promega). AB-PAS staining was performed according to standard protocols. Numbers of Ki-67^+^, TUNEL^+^, and MUC2^+^ epithelial cells were determined by counting foci in 20 low-power fields (magnification ×200).

### Western blotting

Total proteins from colon tissue lysis or cells were extracted using RIPA buffer with protease and phosphatase inhibitors (Roche) and centrifuged for 15 min at 4 °C and 12,000 × *g*. Supernatants were collected. Protein concentrations were measured using a Bradford Protein Assay Kit (Bio-Rad). Equal amounts of proteins from each sample were separated by 10% or 12% sodium dodecyl sulfate-polyacrylamide gel electrophoresis, and transferred onto a nitrocellulose membrane (Millipore). Membranes were blocked by 5% (w/v) non-fat milk powder in Tris-buffered saline containing 0.05% Tween-20 for 1 h at room temperature and immunoblotted overnight at 4 °C with primary antibody. Membranes were washed (three times for 5 min) in PBS with 0.01% Tween 20 (PBST) and incubated with secondary horseradish peroxidase-conjugated antibodies (Cell Signaling) for 60 min at room temperature. Membranes were washed again (three times for 5 min) in PBST and blots were developed using an enhanced chemiluminescence reagent (Thermo Fisher Scientific). The following primary antibodies were used: PRKAR1A (1:1000; Cell Signaling), PRKAR2A (1:1000; Santa Cruz Biotechnology), p-PRKAR2A(ser99) (1:1000; Santa Cruz Biotechnology), p-STAT3(Tyr705) (1:1000; Cell Signaling), STAT3 (1:1000; Cell Signaling), JAK1 (1:1000; Cell Signaling), JAK2 (1:1000; Cell Signaling), p-JAK1(Tyr1034/1035) (1:1000; Cell Signaling), p-JAK2(Tyr1007/1008) (1:1000; Cell Signaling), GAPDH (1:1000; Abcam), and β-actin (1:1000; Cell Signaling).

### RNA analysis

Total RNA was extracted from cell pellets or colon tissues using TRIzol reagent (Invitrogen). RNA was subjected to reverse transcription using the Reverse Transcription Reagent kits (Takara). Real-time PCR was conducted with SYBR Green mix (Applied Biosystems). Reactions were run on a 7500 Fast Real-Time PCR System (Applied Biosystems). Relative quantities of gene transcripts were normalized to *β-actin* transcript levels. Sequences of PCR primers are as follows: 5’-AAATAGGGAAGAAGTGAGCCTC (forward primer for IRF7), 5’-CCCTTGTACATGATGGTCACAT (reverse primer for IRF7), 5’-CTGGCTATTCATCTGGCTGGTCAC (forward primer for OAS2), 5’-GCACCGAGGACACCGCAATC (reverse primer for OAS2), 5’-TCCTGGTGTCCGTGACTAACTCC (forward primer for ISG15), 5’-AGACCGTCCTGGAGCACTGC (reverse primer for ISG15), 5’-TCCTCTTTTTGATAAGGACGCA (forward primer for USP18), 5’-ATCTCATGAGGTGAATGGTCAA (reverse primer for USP18), 5’-TCTGACATCCTGAGCCTCCTTGG (forward primer for APOL9A), 5’-GCCAGTCGGAGCAGCTTCAAC (reverse primer for APOL9A), 5’-CTCTGGCTCAGAATGAAAGGTA (forward primer for ANG4), 5’-TGAAGTTTTCTCCATAAGGGCT (reverse primer for ANG4), 5’-CTGTGTTTCCTTTTCATCCTCG (forward primer for RELMβ), 5’-CTAGTGCAGGAGATCGTCTTAG (reverse primer for RELMβ), 5’-GATCTGGCACCACACCTTCT (forward primer for β-actin), and 5’-GGGGTGTTGAAGGTCTCAAA) (reverse primer for β-actin).

### RNA-seq analysis

Colon tissues collected from WT and *Prkar2a*^−/−^ mice were used for RNA-seq analysis. Whole sequencing process was carried out by Shanghai Personal Biotechnology Co., Ltd. (Shanghai, China). Total RNA was isolated using the Trizol Reagent (Invitrogen Life Technologies), after which the concentration, quality, and integrity were determined using a Nano Drop spectrophotometer (Thermo Scientific). Three micrograms of RNA were used as input material for the RNA sample preparations. Sequencing libraries were generated using the TruSeq RNA Sample Preparation Kit (Illumina). The sequencing library was then sequenced on a Hiseq platform (Illumina). Genes with an adjusted *P* value <0.05 as determined by DE-Seq were designated as differentially expressed.

### Cell lines and culture conditions

Human CRC cell lines (SW480 and RKO) and human normal epithelial cell line CCD841 were cultured in RPMI 1640 medium (Invitrogen), with 10% fetal bovine serum (Gibco) and 1% penicillin–streptomycin (Hyclone) at 37 °C in 5% CO_2_ humidified atmosphere.

### Cell transfections

siRNA that directly target PRKAR2A and scrambled oligonucleotides were purchased from Genepharma Co., Ltd. (Shanghai, China). siRNA-PRKAR2A oligo sequences are as follows: PRKAR2A-Homo-1189 (5’→3’) Sense: GCAGGACUAAAUCAAACAATT. Antisense: UUGUUUGAUUUAGUCCUGCTT. PRKAR2A-Homo-330 (5’→3’) Sense: CCGCCUGACCUCGUCGAAUTT. Antisense: AUUCGACGAGGUCAGGCGGTT. Sequences of Scrambled oligonucleotides are as follows (5’→3’) Sense: UUCUCCGAACGUGUCACGUTT. Antisense: ACGUGACACGUUCGGAGAATT. siRNA transfections were performed using Lipofectamine 2000 reagent (Invitrogen) as described previously^52^. Twenty-four hours after transfection, cells were stimulated with IL-6 or IFN-α or IFN-γ as indicated for 30 min before isolation of total protein.

### Lentiviral transduction

shRNA that specifically targets PRKAR2A or a scramble hairpin were purchased from Donghuan Biotech Co., Ltd. (Shanghai, China). shRNA oligos were all constructed to pLKO.1 vector and plasmids were extracted for lentivirus packaging. Cells were infected with the filtered lentiviral particles in the presence of polybrene and were selected in the presence of puromycin (2 mg/mL) for 1–2 weeks. The knockdown efficiency was determined by western blotting. shRNA sequences designed for knockdown of PRKAR2A are as follows: Forward Oligo Sequence: 5’-CCGGGAGATGTCAAATGCTTAGTTACTCGAGTAACTAAGCATTTGACATCTCTTTTTG-3’. Reverse Oligo Sequence: 5’-AATTCAAAAAGAGATGTCAAATGCTTAGTTACTCGAGTAACTAAGCATTTGACATCTC-3’.

### 16S rDNA sequencing

16S rDNA amplicon libraries were produced from DNA of colon contents and was completed by Shanghai Personal Biotechnology Co., Ltd. (Shanghai, China). Total bacterial genomic DNA samples were extracted using the Fast DNA SPIN Extraction Kits (MP Biomedicals), following the manufacturer’s instructions, and stored at −20 °C prior to further analysis. PCR amplification of the bacterial 16S rRNA gene V3–V4 region was performed using the forward primer 338F (5’-ACTCCTACGGGAGGCAGCA-3’) and the reverse primer 806R (5’-GGACTACHVGGGTWTCTAAT-3’). PCR amplicons were pooled in equal amounts, and pair-end 2 × 300 bp sequencing was performed using the Illumina MiSeq platform with MiSeq Reagent Kit v3 at Shanghai Personal Biotechnology Co., Ltd. (Shanghai, China). The Quantitative Insights Into Microbial Ecology (QIIME, v1.8.0) pipeline was employed to process the sequencing data, as previously described^53^. Sequence data analyses were mainly performed using the QIIME and R packages (v3.2.0).

### Statistical analysis

Data are presented as mean ± SEM. Statistical analysis was performed using GraphPad Prism (GraphPad Software). Statistical significance was calculated using unpaired two-tailed Student’s *t* test. Where more than two groups were compared, one-way analysis of variance (ANOVA) or two-way ANOVA with Bonferroni post hoc test was performed. The significance of survival rate was calculated using log-rank (Mantel–Cox) test. *P* value <0.05 was considered significant.

## Supplementary information

Supplemental figure.
