# Supplementary material for: PRKAR2A deficiency protects mice from experimental colitis by increasing IFN-stimulated gene expression and modulating the intestinal microbiota
Source: Mucosal Immunol. 2021 Aug 4;14(6):1282–94. doi: 10.1038/s41385-021-00426-2 (PMC8528707; doi:10.1038/s41385-021-00426-2)
Supplement: Supplementary file 1 — Supplemental figure. [file 41385_2021_426_MOESM1_ESM.pdf]

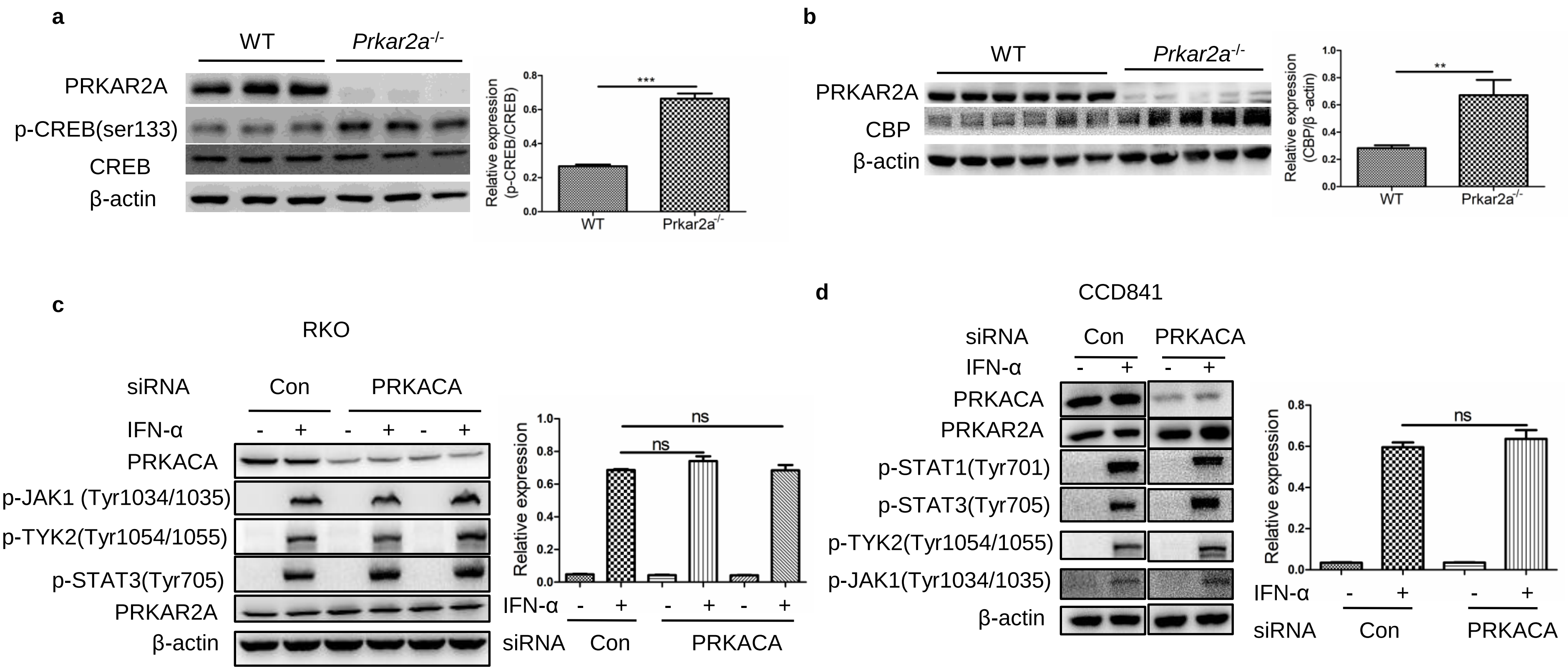

Fig.s1 PKA catalytic subunit does not participate in regulating type I IFN-induced STAT3 activation. Western blot analysis of p-CREB (ser133) (a) and CBP(b) in colon tissues collected from *Prkar2a*$^{-/-}$ and WT mice at day 6 of DSS-induced colitis. Expression of PRKACA was knocked down by siRNA in RKO (c) and CCD841 (d) cell line. The cells were stimulated with or without IFN-α (200ng/ml) for 30min, and the indicated proteins were detected by western blot. Data (a-d) are representative of two independent experiments. Data are presented as mean ± SEM. Student's t-test (a,b,d) or one-way ANOVA (c) was used to do the analysis. **p<0.01, ***p<0.001, ns not significant.

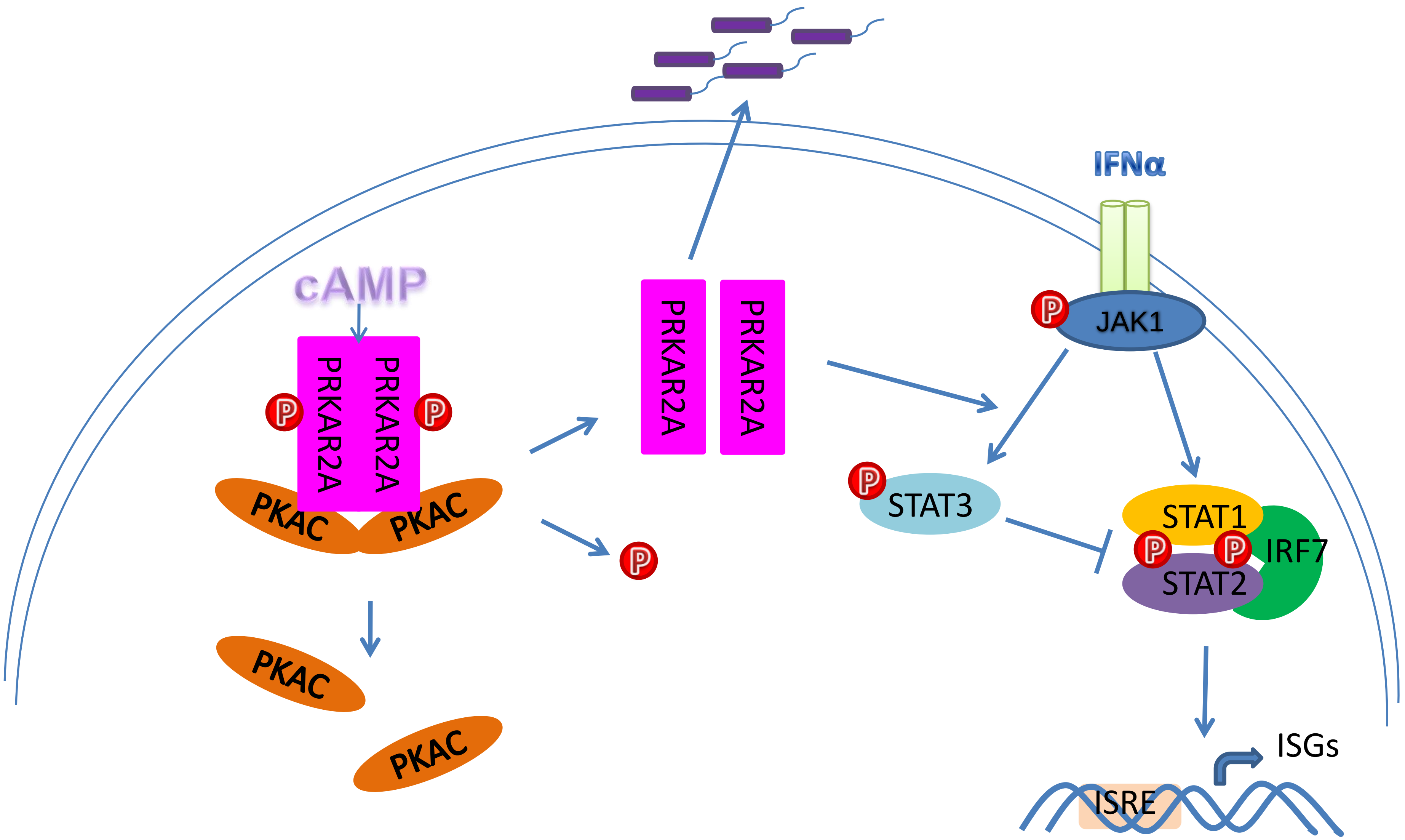

Fig.s2 Schematic diagram of the upstream and downstream signalling of PRKAR2A
